# Chronic Treatment with Curcumin Prevents Vascular Dysfunction in the Aorta of Type 1 Diabetes by Restoring Ca^2+^ Mishandling and Modulating HSP70 Levels

**DOI:** 10.3390/cells14242015

**Published:** 2025-12-17

**Authors:** Swasti Rastogi, Anna Grimm, Brooke Biby, Lucila Mathieu, Brian Trinh, Kenia Pedrosa Nunes

**Affiliations:** Laboratory of Vascular Biology, Department of Biomedical Engineering and Science, Florida Institute of Technology, Melbourne, FL 32901, USA; srastogi2022@my.fit.edu (S.R.); agrimm2020@my.fit.edu (A.G.); bbiby2021@my.fit.edu (B.B.); lmathieu2023@my.fit.edu (L.M.); btrinh2020@my.fit.edu (B.T.)

**Keywords:** curcumin, type 1 diabetes, vascular dysfunction, calcium mishandling, HSP70, extracellular matrix

## Abstract

Vascular Smooth Muscle Cells (VSMC) dysfunction is a major contributor to Type 1 diabetes (T1D)-associated vascular complications. Ca^2+^ is a key messenger responsible for maintaining VSMC tone and function, and alterations in its cytosolic levels are central to diabetes-related vasculopathy. Heat Shock Protein 70 (HSP70), a multifaceted chaperone present intracellularly (iHSP70), regulates vascular reactivity by supporting Ca^2+^ handling, and extracellularly (eHSP70) activates immune signaling. Disruption of eHSP70/iHSP70 balance has been implicated in T1D-associated VSMC dysfunction. Curcumin, a phytochemical found in turmeric, is an emerging therapeutic adjuvant for treating a wide range of pathologies, including diabetes. However, whether curcumin modulates Ca^2+^ dynamics and HSP70 expression, thereby improving VSMC function, in diabetic aorta remains unclear. To investigate this, Streptozotocin-induced diabetic rats (i.p. 65 mg/kg) were treated with curcumin (300 mg/kg) for 28 days. Vascular function was evaluated using wire myography to assess changes in biphasic contraction curve and Ca^2+^ dynamics, while HSP70 was quantified using Western blotting and ELISA. Structural alterations were analyzed by assessing collagen and elastin using Picrosirius staining and fluorescence microscopy. Chronic curcumin treatment improved vascular function by normalizing Ca^2+^ mishandling, restoring the eHSP70/iHSP70 ratio, reducing hypercontractility, and mitigating arterial structural alterations. These findings indicate that curcumin could potentially ameliorate diabetes-related VSMC dysfunction by restoring Ca^2+^ homeostasis and modulating HSP70.

## 1. Introduction

T1D is a metabolic disorder that significantly increases the risk of cardiovascular (CVD) complications and is expected to affect 366 million people worldwide by 2030, highlighting the urgent need to understand its underlying mechanisms [1]. Among these, VSMCs dysfunction is a major contributor to diabetes-associated CVD mortality and morbidity. T1D results from the destruction of pancreatic beta cells, leading to chronic hyperglycemia, further mediating the progression of both microvascular and macrovascular diabetes-associated complications affecting VSMCs [2,3].

VSMCs, which comprise most of the medial layer of the blood vessel, play a crucial role in maintaining proper contraction and relaxation of the vessel. Under physiological conditions, VSMCs maintain a contractile phenotype, particularly in large arteries, ensuring proper tissue perfusion and maintaining the vessel’s structure [4,5]. Among large arteries, the aorta, which is responsible for maintaining pulsatility and withstanding pressure changes, is highly susceptible to hyperglycemia-induced damage [6]. Therefore, the aorta serves as a relevant vessel for studying diabetes-associated VSMCs dysfunction.

Hyperglycemia disrupts VMSCs homeostasis by promoting low-grade inflammation, oxidative stress, changes in contractile responses, and proteins [7,8,9,10,11], as well as extracellular matrix (ECM) alterations involving elastin degradation and increased collagen deposition [12,13,14]. VSMCs play a crucial role in maintaining vascular tone and reactivity, a physiological mechanism highly dependent on the cytosolic Ca^2+^ concentration, which regulates VSMC function. Ca^2+^ mishandling in VSMCs contributes to the progression of diabetic vascular complications [15,16]. Stimulation with α-1 adrenergic receptor agonists such as phenylephrine (PE) elicits a biphasic contraction curve in the aorta comprising a fast phasic component, driven by the release of Ca^2+^ from the sarcoplasmic reticulum (SR) followed by a slow tonic component mediated by the Ca^2+^ influx from the voltage-dependent and -independent channels [17]. In diabetes, this biphasic contraction curve is compromised, indicating Ca^2+^ mishandling in VSMCs [18], leading to impaired contractile responses and changes in the vessel wall, characteristics of diabetic vasculopathies.

One key molecular player in vasculature is HSP70, a chaperone protein critical in both physiological and pathological contexts [18,19,20,21,22]. It is a versatile protein with distinct roles: iHSP70 is present in tissues and exerts anti-inflammatory and cytoprotective effects [23,24], whereas eHSP70, found in circulation, is pro-inflammatory, acting as a Damage Associated Molecular Pattern (DAMP) and activating immune responses by interacting with Toll-Like Receptor 4 (TLR4), an innate immune receptor [25,26]. Emerging studies have identified iHSP70 as a key regulator of vascular reactivity [19], mediating Ca^2+^-dynamics in VMSCs under physiological conditions [20]. Its inhibition impairs both phasic and tonic contraction in the aorta [19] with sex-dependent effects [21] under physiological conditions. In contrast, in aging, HSP70 blockade selectively affects tonic contraction [22], highlighting its regulatory role in Ca^2+^-dependent vascular function. Maintaining a balance between both iHSP70 and eHSP70 is crucial to vascular homeostasis, and disruption of this balance is a feature of VSMC dysfunction and low-grade inflammation in diabetes [18,27].

Likewise, structural alterations in the aortic wall contribute to the diabetes-associated vasculopathy. The aortic ECM is composed of two main proteins- collagen and elastin, which provide mechanical strength and elasticity, allowing the vessel to recoil and withstand the large volume changes [28]. Diabetes causes ECM remodeling characterized by collagen deposition, elastin reduction, and fragmentation, leading to decreased vascular compliance [12,13,14]. Disrupted Ca^2+^ signaling and HSP70 have also been implicated in promoting these alterations [29,30].

Given the growing interest in natural compounds as therapeutic interventions, curcumin—a polyphenol derived from *Curcuma longa*—has emerged as a potential therapeutic agent with well-documented pharmacological benefits, including antioxidant, anti-inflammatory, anti-atherogenic, cardiovascular, and neuroprotective effects [31,32,33,34]. In diabetes, curcumin has been shown to lower glucose and improve beta-cell activity [34,35,36,37]. It enhances VSMC function [38] and mitigates ECM disruption by reducing collagen deposition in arteries [39]. Additionally, curcumin affects L-type Ca^2+^ channels in neuronal cells [40], suggesting the possibility that it may modulate disrupted Ca^2+^ dynamics in the vasculature. Interestingly, curcumin has also been shown to upregulate HSP70 expression in various cell types [41,42], highlighting a potential mechanism for improving VSMC function. However, whether curcumin stabilizes Ca^2+^ mishandling, restores the eHSP70/iHSP70 ratio balance, and prevents structural alterations in the aorta of Type 1 diabetic animals remains unclear. Therefore, in this study, we aimed to investigate the effects of chronic curcumin treatment on vascular contractility, Ca^2+^ dynamics, HSP70 expression, and alterations in the vessel wall of the aorta in T1D rats.

## 2. Materials and Methods

### 2.1. Animals

All animal procedures were followed in accordance with National Institutes of Health guidelines for the Care and Use of Laboratory Animals. This protocol was reviewed and approved by the Institutional Animal Care and Use Committee at the Florida Institute of Technology (Protocol No. 2023-01, approval date: 26 September 2023). Male Sprague-Dawley rats, approximately 6–7 weeks old (n = 24), were obtained from Taconic Biosciences. The animals were kept in a controlled environment at room temperature with a consistent 12 h light-dark cycle and unrestricted access to water and food. The body weight and glucose levels of all rats were monitored throughout the treatment period, up to 28 days. A commercially available Accu-Chek glucose monitoring kit (Roche, Boston, MA, USA) was used to monitor glucose levels in all fasting animals. Animals were sacrificed by exsanguination at 12 weeks under anesthesia, isoflurane (5% in 100% O_2_). All the procedures were performed in compliance with ARRIVE guidelines [43] and under the supervision of a veterinarian. All efforts were made to reduce the number of animals used in the study and to ensure reduced pain and distress, in accordance with the 3Rs (Replacement, Reduction, and Refinement).

### 2.2. STZ-Induced Diabetes

After a 48 h period of acclimation, the animals were randomly divided into diabetic and non-diabetic groups. Diabetes was induced by administering Streptozotocin (65 mg/kg; ThermoFisher Scientific, Waltham, MA, USA, No. J61601.ME) intraperitoneally. The control animals were injected with an equal volume of citrate buffer (pH 4.5). The animals were confirmed diabetic with glucose levels above 250 mg/dL.

### 2.3. Curcumin Treatment

Animals were randomly divided into four groups to receive the curcumin treatment 300 mg/kg by body weight daily via oral administration. The established groups were: (1) control animals, (2) control animals treated with curcumin, (3) diabetic animals, and (4) diabetic animals treated with curcumin. Following the previously described protocol [44], curcumin (ThermoFisher Scientific, Waltham, MD, USA, No. 218580500) was administered daily for 28 consecutive days. Animals from the treatment group were given curcumin mixed with peanut butter (PB) by voluntary consumption to minimize distress and reduce the risk of injury associated with oral gavage. The curcumin-PB mixture was administered at the beginning of the light-dark cycle, after a 3 h fasting period. Standard chow was provided 2 h after the curcumin consumption. The animals were weighed every week to account for any changes in body weight. At the end of the treatment, animals were anesthetized (4% isoflurane in 100% O_2_), and blood was collected from the abdominal aorta using a BD Vacutainer Blood Collection Tube. Blood samples were kept on ice for approximately 30 min before being centrifuged at 3500× *g* for 25 min. The serum supernatant was collected, snap-frozen in liquid nitrogen, and stored at −80 °C for further analysis of extracellular eHSP70 by Western blotting and ELISA. From the same animal, the aorta was extracted and cleaned in enriched Physiological Salt Solution (PSS) under the microscope for subsequent analysis.

### 2.4. Functional Studies

Aortic rings (2 mm) were obtained from the thoracic aorta, cleaned of all surrounding adipose tissues, and mounted in a multi-wire Myograph system DMT620M (Danish MyoTechnology, Aarhus, Denmark) with a resting tension of 30 mN. Each myograph chamber was filled with PSS (in mmol/L: 130 NaCl, 4.7 KCl, 1.18 KH_2_PO_4_, 1.18 MgSO_4_*7H_2_0, 14.9 NaHCO_3_, 5.6 Dextrose, 1.56 CaCl_2_*H_2_O, 0.026 EDTA) and aerated with carbogen (95% CO_2_ and 5% O_2_) and maintained at 37 °C, to provide ideal physiological conditions. The rings were allowed to equilibrate for 60 min, during which the PSS was replaced every 15 min from each chamber. The resting tension was adjusted each time to 30 mN. To assess the viability of rings, the PSS was replaced with a 120 mM KCl. The rings contracting to KCl were considered viable, with a tension of more than 50% of the baseline. Then the rings were washed, allowed to return to the resting tension, and processed as follows.

#### 2.4.1. Concentration-Response Curve and Time-Force Curve

A cumulative concentration-response curve to PE (1 nmol/L to 100 μmol/L) was obtained in aortic rings with intact endothelium to understand the contractile response.

In another set of experiments, after washing, equilibration, and verifying viability, the aortic rings were challenged with a single dose of PE (10 μmol/L), and the isometric force development was continuously monitored and recorded for 10 min. The biphasic contraction components- phasic (rapid) and tonic (sustained) contraction were evaluated upon PE stimulation of aortic rings. The phasic part was calculated by subtracting the baseline force from the peak force observed during this rapid increase. The tonic component was calculated by subtracting the baseline force from the force measured during the stabilized part of this sustained contraction.

#### 2.4.2. Calcium Protocol

To further understand the calcium dynamics underlying each contraction phase, a separate Ca^2+^ mobilization protocol was performed. The aortic rings, 2 mm in length, were mounted on a wire myograph, equilibrated and checked for viability. This protocol was performed with and without exogenous Ca^2+^. The PSS was replaced with a Ca^2+^-free PSS containing 1 mmol/L EGTA at time zero (t_0_). After 3 min, the rings were challenged with PE (10 μmol/L), and the generated force was measured for 10 min. After this, Ca^2+^ was restored in the chamber to reestablish the original PSS concentration (1.56 mmol/L), and the resulting force was recorded for an additional 15 min. The Ca^2+^ efflux representing generated force in response to Ca^2+^ release from the SR was calculated as the peak force following addition of PE minus the baseline force at t_0_ in the Ca^2+^-free PSS. The Ca^2+^ influx, reflecting Ca^2^ release from plasma membrane channels, was calculated as the peak force at the end of the curve minus the baseline force in PSS before exogenous calcium. This protocol enables the indirect assessment of cytosolic Ca^2+^ levels as a function of change in development of force.

### 2.5. Western Blot

Aortic tissue was homogenized in Tissue Extraction Protein Reagent (ThermoFisher Scientific, Waltham, MD, USA, No. 78510) and mixed with Protein Inhibitor Cocktail (Sigma Aldrich, St. Louis, MO, USA, No. P3840). The protein concentration in the homogenized samples was determined using a BCA Protein Assay kit (ThermoFisher Scientific, Waltham, MD, USA, No. 23225). The serum samples were diluted to 1:10 in the lysis buffer. Subsequently, the total of 10 ug protein from (aorta) and 10 uL from (serum) was loaded onto a 10% SDS-PAGE gel, followed by transfer onto a nitrocellulose membrane using a semi-dry blotting system. Membranes were stained with Ponceau stain to confirm uniform protein transfer, imaged, and washed with Tris buffer containing 1% Tween-20. The membranes were then incubated at room temperature for 1 h in blocking buffer containing 5% non-fat dry milk in TBST to block non-specific binding. Following this, the membrane was incubated with the primary antibody for HSP70 (1:1000 dilution in TBST containing 0.5% BSA, Cell Signaling Technology, Danvers, MA, USA, No. 4872S) at 4 °C overnight. The membrane was washed with TBST and again incubated again with the secondary antibody (dilution 1:10,000 in TBST, Cell Signaling Technology, Danvers, MA, USA, No. 7074S). Next, the membranes for aortic HSP70 were stained with 700-Revert Total Protein Stain (Li-COR Biosciences, Lincoln, NE, USA, No. 926-110116) and later normalized to total protein, while serum HSP70 was normalized to ponceau. The band detection was performed using SuperSignal West Femto Substrate (ThermoFisher Scientific, Waltham, MD, USA, No. 34095), and bands were visualized using Chemidoc MP Imaging System (Bio-Rad, Hercules, CA, USA) using auto-exposure function, kept consistent throughout. Densitometric analysis was performed in ImageJ version 1.54p (NIH, Bethesda, MD, USA). The band quantification was done separately for each band and normalized to their corresponding total protein or ponceau. No background subtraction was done for the quantification.

### 2.6. ELISA

Levels of circulating eHSP70 in serum were detected using a commercially available AMP’D High Sensitivity HSP70 ELISA Kit (Enzo Life Science, Farmingdale, NY, USA, ENZ-KIT-101) as per the manufacturer’s instructions.

### 2.7. Elastin Characterization

The aortic tissue was embedded in Optimal Cutting Temperature (OCT) to support and maintain its structural integrity and then frozen to obtain a 10 μm-thick on a cryostat. Subsequently, aortic sections were washed with 1×PBS to remove any remaining OCT. The rings were then mounted with Vectashield Antifade Mounting Medium (Vector Laboratories, Newark, CA, USA, H-1000), and fluorescence with excitation at 488 nm was captured and images were obtained at both 4× and 10× magnification. Autofluorescence intensity was measured in arbitrary units using ImageJ software version 1.54p (NIH, Bethesda, MD, USA) with the background removed from the images (4× magnification) and was quantified using the threshold function. The number of laminae was manually counted (10× magnification). Representative images were adjusted for brightness and contrast using consistent settings across all samples.

### 2.8. Collagen Detection

Collagen levels in 10 μm-thick aortic ring sections were measured using a Picrosirius staining commercial kit (Abcam, Waltham, MA, USA, ab150681), following the manufacturer’s instructions. After 24 h, images were captured using a Zeiss AxioSkop-2 MOT microscope (10× magnification; Zeiss, Jena, Germany). ImageJ software version 1.54p (NIH, Bethesda, MD, USA) was used to analyze the deposition of collagen, expressed as the percentage of positive deposition (% positive area; red staining) in the total vessel (media layer + adventitia) compared to the media layer. Adjustments for brightness and contrast were made to the images, maintaining consistent settings across all samples.

### 2.9. Statistical Analysis and Data Normalization

All the data, analysis, and graph generation were conducted using GraphPad Prism version 10.4.1 (GraphPad Software, San Diego, CA, USA). Data normality was assessed using the Shapiro–Wilk test. For the dataset significant for normality, group comparisons were conducted using one-way ANOVA, followed by Bonferroni’s post hoc test. Results are expressed as means ± SEM. For data that did not fit the normality criteria, the Mann–Whitney U test was performed, and data is presented as median with Interquartile range (IQR). Outliers were identified using Grubb’s test. A *p*-value of <0.05 was considered statistically significant. The sample size (n) denotes the number of rats in each group.

For dose–response and time-force curves, we normalized PE-induced contraction values to the maximum contraction elicited by KCl to account for the receptor-mediated mechanism. No normalization to KCl was performed in the Calcium protocol, as it specifically evaluates the force generated by SR Ca^2+^ release and extracellular Ca^2+^ influx.

The ratio eHSP70/iHSP70 was calculated by dividing the densitometric value of eHSP70 by that of iHSP70. The H-index, an indirect marker of low-grade inflammation, was calculated by dividing the eHSP70/iHSP70 ratio of diabetic animals by the ratio of controls, which was set to 1 as previously described [18,45].

## 3. Results

### 3.1. Curcumin Mitigates Hypercontractility in the Diabetic Aorta

**Figure 1:**
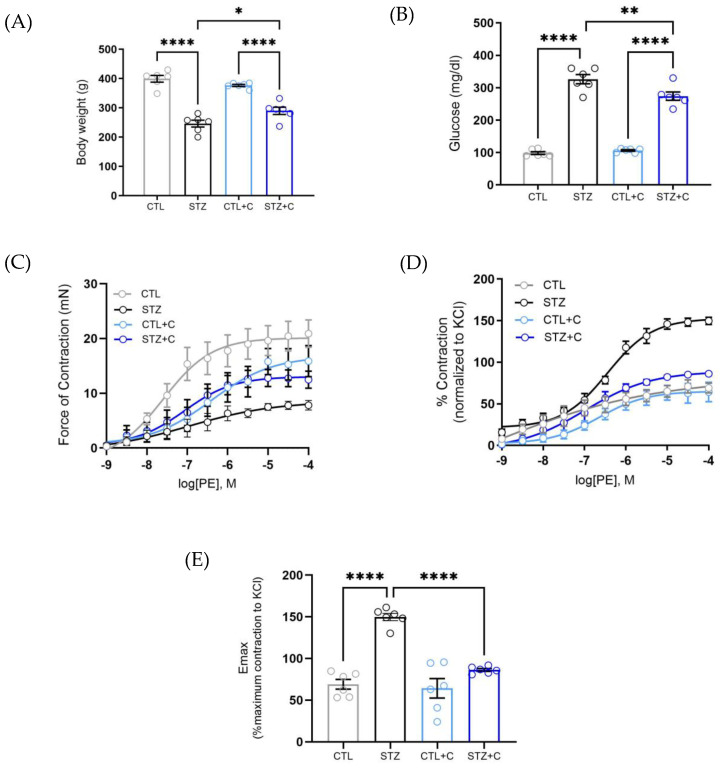
Effects of chronic curcumin treatment on animal profile and Phenylephrine (PE) − induced dose–response curve in the isolated aortic rings. (**A**) Body Weight (g). (**B**) Blood Glucose levels (mg/dL). (**C**) Dose–response curve to Phenylephrine (PE, 1 nmol/L to 100 μmol/L). (**D**) % Contraction normalized to KCl. (**E**) The maximum response (Emax). Groups include control (gray circles), diabetic (black circles), control + curcumin (light blue circles), and diabetic + curcumin (dark blue circles). Data are presented as means ± SEM. Statistical analyses were performed using one−way ANOVA, followed by Bonferroni’s post hoc; n = 6 per group. *p*-values indicating significance * *p* < 0.05, ** *p* < 0.01, **** *p* < 0.0001.

Before assessing vascular function, the animal profile was characterized. Diabetic rats exhibited reduced body weight and elevated blood glucose levels compared to non-diabetic controls. Chronic curcumin treatment significantly improved both body weight and glucose levels in the diabetic-treated rats, though they remained hyperglycemic (Figure 1A,B).

Hypercontractility is a well-described feature of the aorta in diabetic models [17,28]. To investigate whether curcumin mitigates PE-induced hypercontractility, we assessed its effects on aortic rings. Diabetic aortic rings demonstrated a rightward shift in the dose–response curve to phenylephrine, at a lower proportion, which was corrected upon curcumin treatment (Figure 1C). When the force values from α-1 adrenergic contraction were normalized to maximum response elicited by KCl, to account for receptor mediated mechanism, diabetic aorta displayed hypercontractility compared to controls (Figure 1D). Chronic curcumin treatment reduced this increased vasoconstriction in diabetic aortas (Figure 1D), also significantly attenuating the maximum contractile response (Emax; Figure 1E) elicited by PE in the treated rats.

### 3.2. Curcumin Treatment Attenuates Phasic and Tonic Components of Vascular Contraction in the Diabetic Aorta

**Figure 2:**
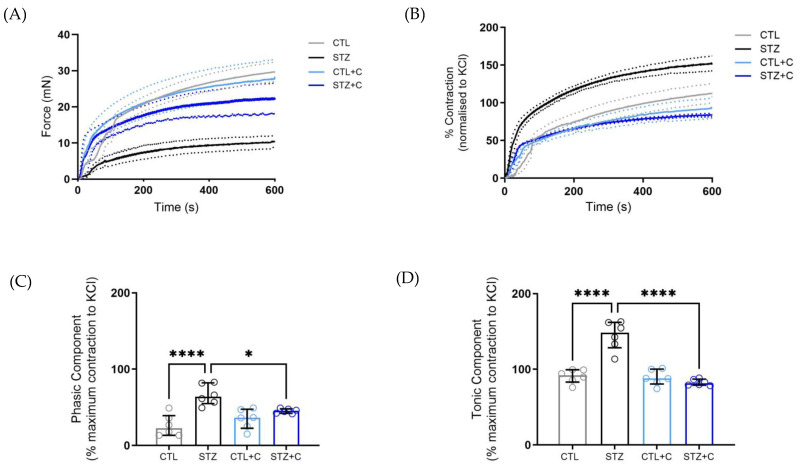
Effects of curcumin treatment on the biphasic contraction in the aorta of diabetic rats. (**A**) Representative time vs. force curve of the aorta stimulated with a single dose of Phenylephrine (PE, 10 μmol/L). The force (mN) generated was analyzed for a period of T = 600 s. (**B**) Representative time vs. force curve of the aorta normalized to KCl. (**C**) Phasic Component. (**D**) Tonic Component. Groups include control (gray circles), diabetic (black circles), control + curcumin (light blue circles), and diabetic + curcumin (dark blue circles). The dotted lines represent error bars. Data is expressed as median with IQR. Statistical analyses were performed using Mann−Whiney U test; n = 6 per group. *p*-values indicating significance * *p* < 0.05, **** *p* < 0.0001.

We next evaluated whether curcumin affects force development in response to a single dose of an alpha-1 adrenergic agonist, generating a biphasic contraction curve that reflects a two-phase increase in cytosolic calcium. The diabetic rings displayed diminished force development compared to the control and treated groups (Figure 2A). When normalized by KCl, the diabetic rings exhibit enhanced force development, characterized by an increase in both the phasic and tonic components of the contraction curve (Figure 2A–C). However, the force development generated was significantly reduced with curcumin treatment, restoring contraction to control levels in both components of the curve in the treated diabetic aortic rings (Figure 2A–C).

### 3.3. Chronic Treatment with Curcumin Restores Altered Calcium Dynamics in the Diabetic Aorta

**Figure 3:**
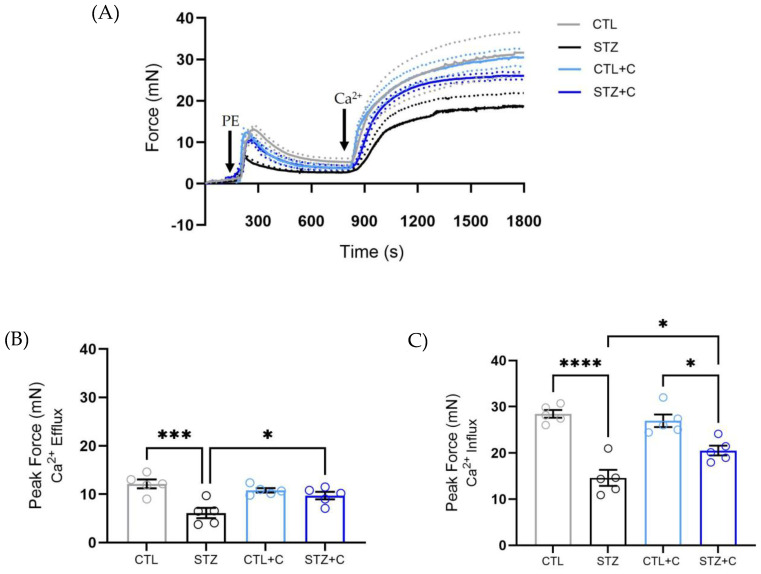
Effect of curcumin on disrupted Calcium dynamics in the aorta of diabetic rats. (**A**) Representative time vs. force curves of the aorta during Ca^2+^ protocol (arrow indicates stimulation of aortic rings with Phenylephrine and exogenous Ca^2+^). (**B**) Peak Force in response to Phenylephrine (PE, 10 μmol/L), showing Ca^2+^ efflux. (**C**) Peak Force in response to Ca^2+^ influx in the aorta when Ca^2+^ is restored. Groups include control (gray circles), diabetic (black circles), control + curcumin (light blue circles), and diabetic + curcumin (dark blue circles). The dotted lines represent error bars. Statistical analyses were performed using one−way ANOVA followed by Bonferroni’s post hoc test; n = 5 per group. *p*-values indicating significance * *p* < 0.05, *** *p* < 0.001, **** *p* < 0.0001.

Since, Ca^2+^ is a key signaling molecule regulating VSMCs’ contraction, we subsequently investigated effect of curcumin on underlying Ca^2+^ mobilization to corroborate our observations from biphasic contraction curve. To examine this, aortic rings were stimulated with PE in Ca^2+^-free PSS to assess Ca^2+^ release from SR. Next, the extracellular Ca^2+^ was reintroduced to assess Ca^2+^ influx from plasmalemmal channels. Diabetic rats showed reduced force generation in response to both Ca^2+^ release from SR following transient contraction and Ca^2+^ influx, indicating impaired Ca^2+^ handling (Figure 3A–C). Notably, chronic curcumin treatment significantly improved force development in both Ca^2+^ efflux (Figure 3B) and influx, (Figure 3C), indicating restored Ca^2+^ homeostasis (Figure 3A–C).

### 3.4. Curcumin Modulates HSP70 Expression in the Diabetic Aorta

**Figure 4:**
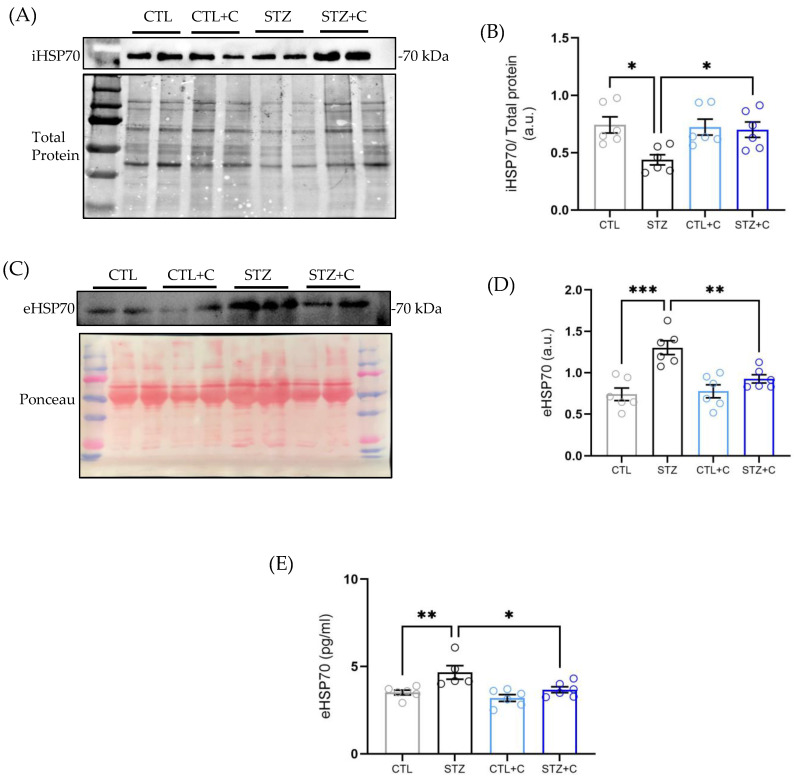
Effect of curcumin on HSP70 expression in diabetic aorta. (**A**) Blot showing expression of iHSP70 in the aortic tissue. (**B**) iHSP70 densitometry. (**C**) levels of eHSP70 in the serum by Western blot. (**D**) eHSP70 densitometry. (**E**) eHSP70 serum levels by ELISA. Groups include control (gray circles), diabetic (black circles), control + curcumin (light blue circles), and diabetic + curcumin (dark blue circles). Data is expressed as mean ± SEM. Statistical analyses were performed using one−way ANOVA followed by Bonferroni’s post hoc test; n = 5−6 per group, a.u.: arbitrary units. *p*-values indicating significance * *p* < 0.05, ** *p* < 0.01, *** *p* < 0.001.

HSP70, the molecular chaperone mechanistically supports Ca^2+^ handling in the isolated aorta while eHSP70 acts as DAMP, triggering immune responses. Diabetes disrupts this balance between intracellular and extracellular HSP70, characterized by reduced aortic iHSP70 and elevated eHSP70 levels leading to VSMC dysfunction under T1D [18]. To investigate whether curcumin modulates HSP70 expression, iHSP70 levels were assessed in aortic rings, and eHSP70 was measured in the serum from diabetic and control rats. Interestingly, curcumin treatment increased the aortic iHSP70 expression (Figure 4A,B) and reduced circulating eHSP70 levels in diabetic rats quantified by both Western blotting (Figure 4C,D) and ELISA (Figure 4E), indicating that curcumin helps to restore HSP70 homeostasis.

### 3.5. Curcumin Restores the eHSP70/iHSP70 Ratio in the Diabetic Aorta

**Figure 5:**
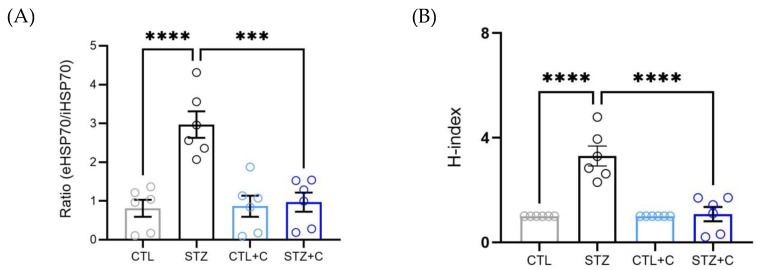
Curcumin’s effect on the ratio eHSP70-to-iHSP70 and H-index in diabetic aorta. (**A**) eHSP70−to−iHSP70 ratio, and (**B**) H-index. Groups include control (gray circles), diabetic (black circles), control + curcumin (light blue circles), and diabetic + curcumin (dark blue circles). Data is expressed as mean ± SEM. Statistical analyses were performed using one−way ANOVA followed by Bonferroni’s post hoc test; n = 6 per group. *p*-values indicating significance *** *p* < 0.001, **** *p* < 0.0001.

While previous studies have reported elevated eHSP70/iHSP70 ratio in diabetic models [18,29], whether the curcumin impacts it remains unclear. Corroborating previous findings, we observed elevated eHSP70/iHSP70 ratio in the diabetic rats, which was restored to levels like controls following curcumin treatment (Figure 5A). Additionally, H-index, an indirect marker of low-grade inflammation derived by dividing the ratio in diabetic rats by the ratio in controls, set to 1 as previously shown [18], was elevated in diabetic rats and significantly reduced by curcumin (Figure 5B), further supporting anti-inflammatory effects of curcumin.

### 3.6. Treatment with Curcumin Prevents Elastin Fragmentation and Collagen Deposition in the Diabetic Aorta

**Figure 6:**
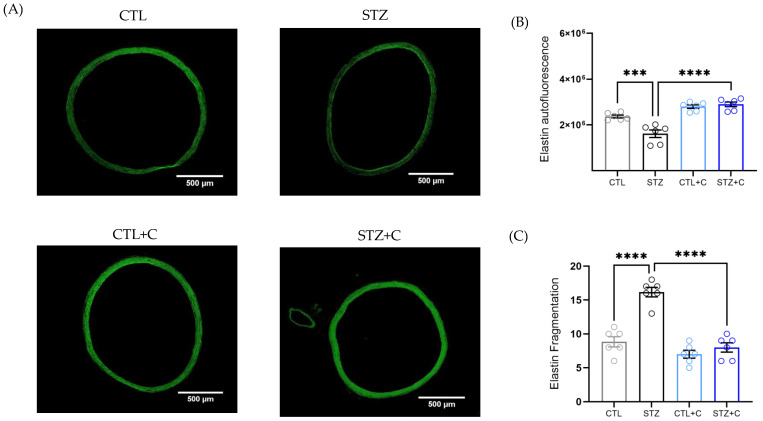

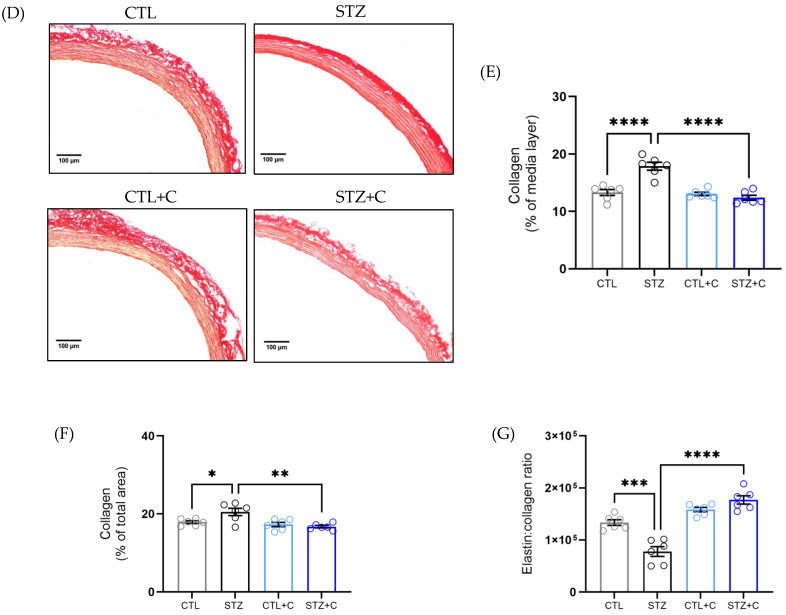
Effect of Curcumin treatment on elastin and collagen in the aorta of diabetic rats. (**A**) Representative images of the aorta showing elastin autofluorescence (green; scale: 500 μm). (**B**) Elastin autofluorescence. (**C**) Elastin fragmentation. (**D**) Representative images of the aorta stained with Picrosirius stain for collagen (red; scale: 100 μm). (**E**) Collagen percentage of medial layer, (**F**) Collagen percentage of total layer. (**G**) Elastin-to-Collagen ratio. Groups include control (gray circles), diabetic (black circles), control + curcumin (light blue circles), and diabetic + curcumin (dark blue circles). Data are presented as means ± SEM. Statistical analyses were performed using one−way ANOVA followed by Bonferroni’s post hoc test; n = 6 per group. *p*-values indicating significance. * *p* < 0.05, ** *p* < 0.01, *** *p* < 0.001, **** *p* < 0.0001.

Elastin and collagen are the two essential extracellular matrix (ECM) components in the large arteries, maintaining vessel wall structure and mechanical properties. In diabetes, disruption of their organization contributes to VSMCs’ dysfunction. To evaluate whether curcumin mitigates these ECM alterations, elastin and collagen in the aorta were quantified indirectly. Diabetic aorta exhibited reduced elastin autofluorescence, accompanied by increased fragmentation of elastin, indicative of fenestration, along with increased collagen deposition both the medial layer and the total vessel (Figure 6A–F). Curcumin treatment effectively restored elastin levels to those of controls, reduced the fragmentation (Figure 6A–C) and attenuated the collagen deposition (Figure 6D–F). Importantly, the decreased elastin: collagen ratio in the diabetic aorta was restored following curcumin treatment, thereby maintaining vessel wall structure (Figure 6F).

## 4. Discussion

Curcumin, a natural polyphenol compound extracted from *Curcuma longa* (turmeric), has been widely studied due to its broad range of biological activities, including its potential use for treating cardiovascular disorders [32,46,47,48]. However, whether curcumin can exercise vasculo-protective effects under T1D, specifically by attenuating VSMC hypercontractility, stabilizing Ca^2+^ dynamics, and modulating HSP70 expression, while preserving aortic structure in T1D, remains unclear.

As hyperglycemia drives VSMC dysfunction in diabetes, and curcumin has been shown to attenuate glucose levels [34,35,36,37], it is imperative to investigate treatments that promote vascular protection. Accordingly, we evaluated the effect of curcumin on glucose levels and body weight in STZ-induced diabetic rats. Consistent with previous reports, chronic curcumin treatment improved body weight and reduced elevated blood glucose (Figure 1A,B), supporting its anti-diabetic effects. Notably, despite the improvement in glucose levels, curcumin-treated diabetic rats remained hyperglycemic. These effects are likely attributed to curcumin’s ability to enhance metabolic homeostasis by reducing inflammation and oxidative stress, independent of changes in appetite. Conversely, reports with curcumin show no significant changes in blood glucose, body weight, blood pressure, or appetite [49,50,51].

Diabetes induces vascular alterations, impairing Ca^2+^ handling and leading to phenotypic changes in VSMCs, which results in a shift from a contractile to a synthetic state [52,53]. This shift contributes to hypercontractility [54,55], driven by hyperglycemia and vessel wall stiffness [56,57], which leads to reduced compliance. Past studies have reported enhanced vasoconstriction to PE in both mesenteric arteries and the aorta of diabetic rats [54,55], although some have reported attenuated responses [58,59]. Our results confirmed that the diabetic aorta exhibits elevated contractile responses to PE as observed in increased maximum response (Emax), indicating hypercontractility (Figure 1C–E). Importantly, curcumin significantly attenuated this effect (Figure 1C–E), reflecting its capability to restore vascular function. Considering diabetes disrupts Ca^2+^ signaling, we next investigated whether curcumin affects the biphasic contraction curve. Both phasic and tonic components of the curve were elevated in the diabetic aorta (Figure 2C,D), showing dysregulated Ca^2+^ dynamics, which were restored following chronic curcumin treatment. This demonstrates its potential to correct impaired contractile response and Ca^2+^ mishandling in the aorta. To further corroborate our findings from biphasic contraction, we assessed curcumin’s effect on Ca^2+^ mobilization in the presence and absence of extracellular Ca^2+^. In the absence of exogenous Ca^2+^, the diabetic aorta exhibited reduced force development to PE (Figure 3A,B), indicating impaired Inositol 1,4,5-triphosphate Receptor (IP3R)-mediated Ca^2+^ release as previously described in other studies [59,60]. Curcumin restored this response, suggesting improved IP3R-mediated signaling. Upon reintroduction of extracellular Ca^2+^, the diabetic aorta displays impaired force development, reflecting disrupted Ca^2+^ influx through voltage-dependent and -independent channels [61,62]. This response was improved by curcumin, corroborating observations in superior mesenteric arteries [63]. The protocol used here is an indirect measure of Ca^2+^ mobilization, as changes in force development reflect alterations in Ca^2+^ handling. Together, these findings suggest that curcumin restores Ca^2+^ dynamics, thereby contributing to the improvement of vascular function in T1D.

A potential regulator of Ca^2+^ dynamics in VSMCs is HSP70, and curcumin has been shown to upregulate HSP70 expression in various cell types [41,42]. HSP70 is a highly conserved family of molecular chaperones crucial for maintaining protein homeostasis. In humans, 13 genes encode proteins within this family, which includes both inducible and constitutive isoforms that regulate protein folding and multiple signaling pathways [64,65]. In this study, we use HSP70 to denote the entire family, unless specified otherwise. This protein supports vascular reactivity under physiological conditions by mediating phasic contraction through crosstalk with IP3R-mediated Ca^2+^ release, and tonic contraction via voltage-independent Ca^2+^ influx [19,20]. Inhibition of HSP70 leads to impaired force development in response to Ca^2+^ mobilization in a sex-based manner under physiological conditions, as well as with increasing age [21,22].

Initially, HSP70 was considered a cytoplasmic protein with intracellular roles; however, evidence suggests that it can also be released into the extracellular space [66]. The iHSP70 within tissues primarily acts as an anti-inflammatory agent [23,24] and modulates Ca^2+^ homeostasis in VSMCs [20]. In contrast, eHSP70 can act as a DAMP, binding to TLR4 and activating pro-inflammatory responses [18,25,26], demonstrating HSP70’s antagonistic actions depending on its localization. Therefore, a balance between iHSP70 and eHSP70 is imperative in determining whether HSP70 will have protective or detrimental effects. HSP70 has long been implicated in diabetes and is tissue specific [18,25,27,67]. In the aorta, reduced iHSP70 and elevated levels of eHSP70 have been reported in T1D [18], with disruptions also observed in type 2 diabetes (T2D) [27]. Corroborating this, we showed decreased aortic iHSP70 alongside elevated eHSP70 levels in the diabetic rats compared to controls (Figure 4A,B). Notably, curcumin treatment reversed these imbalances, upregulating iHSP70 expression (Figure 4A,B) and reducing eHSP70 levels, as confirmed by Western blotting and ELISA (Figure 4C–E). Furthermore, diabetic rats exhibited a significantly elevated eHSP70/iHSP70 ratio and H-index (Figure 5A,B), indicating a shift towards a pro-inflammatory state, consistent with the chaperone balance hypothesis, which proposes that due to the opposing nature of iHSP70 and eHSP70, an imbalance between them reflects inflammatory status in pathological states, including diabetes [45,68]. Curcumin treatment normalized the eHSP70/iHSP70 ratio and H-index (Figure 5A,B). To the best of our knowledge, this is the first evidence of its effect, demonstrating that curcumin restores HSP70 homeostasis in diabetes.

Diabetes appears to affect the aorta more than other elastic arteries [6]. The aorta, a major conduit artery rich in elastin, is critical in maintaining pulsatile blood flow and hemodynamics (Windkessel function) [57]. Any functional or structural alterations in the aorta significantly affect the downstream blood flow, leading to end-organ damage. Aortic compliance depends on the balance between two ECM components: elastin, which provides the ability to recoil, allowing it to adapt to changing pulsatile flow, and collagen, which imparts tensile strength. Large arteries exhibit a higher elastin-to-collagen ratio than distal arteries, with the elastin content gradually decreasing with distance from the heart [69].

ECM alterations in diabetes are characterized by increased collagen and reduced elastin [12,13,14], which are not uniform across different vascular beds and are also sex -dependent [70]. For example, in the T2D model, aortic elastin-to-collagen mRNA ratio is reduced, with an opposite trend observed in the coronary artery [71], highlighting the need for potential therapeutics that preserve aortic wall structure. In addition, the composition of aortic ECM is influenced by multiple mechanisms, including increased collagen synthesis via transforming growth factor type-β1 (TGF-β1) mediated by HSP70-TLR4 signaling, and dysregulated Ca^2+^ dynamics via Matrix Metalloproteinases (MMPs), which are upregulated in diabetes [29,30]. Diabetes-related Ca^2+^ fluctuations may affect MMP activity and promote ECM degradation, suggesting that Ca^2+^ dysregulation and HSP70 may be involved in ECM alterations. In our study, diabetic aorta exhibited reduced elastin autofluorescence, increased fragmentation, and collagen accumulation in both the medial layer and the total vessel, resulting in a reduced elastin-to-collagen ratio that is reflective of stiffer vessels (Figure 6A–G). Notably, curcumin treatment prevented all these alterations (Figure 6A–G). A similar pattern has been reported in aged mice, where curcumin normalizes aortic collagen, but without significant changes in elastin [72].

As limitations of this study, we acknowledge that Ca^2+^ mobilization was indirectly measured, and techniques such as patch clamp, would provide more direct evidence of Ca^2+^ handling in the vasculature. Similarly, the use of atomic force microscopy could further validate our ECM results. While the H-index is a marker of inflammation, quantification of inflammatory cytokines would strengthen our findings. Despite these limitations, this study provides a comprehensive overview of curcumin’s functional and structural effects on the aorta under diabetes, suggesting this compound has a beneficial outcome by preventing vascular dysfunction.

Beyond these experimental limitations, curcumin itself presents several challenges that limit its therapeutic use, including its poor solubility and rapid elimination [73]. Moreover, curcumin interacts with a wide range of proteins, including kinases, reductases, and inflammatory molecules, as well as binds to DNA/RNA, thereby modulating multiple signaling pathways [74]. This multitarget and pleiotropic behavior of curcumin is advantageous, owing to its broad pharmacological properties, but also makes it difficult to attribute curcumin’s promising effects to a single and specific mechanism.

Together, our findings demonstrate that curcumin improves VSMC function in diabetes by preventing Ca^2+^ mishandling. Although curcumin-treated diabetic animals remained hyperglycemic, the normalized vascular function observed may involve glucose-independent mechanisms, with HSP70 potentially mediating this effect. Mechanistically, in non-vascular cell types, curcumin upregulates HSP70 expression through the activation of Heat-shock factor 1 (HSF-1)- mediated transcription, inhibition of p38 mitogen-activated protein kinase (p38-MAPK), and suppression of pro-inflammatory cytokines [41,42]. While these mechanisms have not been directly observed in the vasculature, future studies are needed to better understand the HSP70-dependent pathways that drive these vasculo-protective effects associated with curcumin under diabetes.

## 5. Conclusions

Overall, chronic curcumin treatment improved vascular function by attenuating hypercontractility observed in the aorta of the T1D model, normalizing Ca^2+^ mishandling as demonstrated in our Ca^2+^ protocol, restoring the eHSP70/iHSP70 balance by upregulating iHSP70 while reducing eHSP70, and mitigating arterial structural alterations. Although curcumin exhibits limited bioavailability and is a multifaceted natural compound, our findings highlight that long-term use of curcumin minimizes both the functional and structural complications associated with T1D-induced vascular dysfunction, thereby favorably affecting most aspects of diabetes.

## Data Availability

The original contributions presented in the study are included in the article; further inquiries can be directed to the corresponding authors.

## Abbreviations

The following abbreviations are used in this manuscript:

VSMCs	Vascular Smooth Muscle Cells
T1D	Type 1 Diabetes
STZ	Streptozotocin
iHSP70	Intracellular Heat Shock Protein 70
eHSP70	Extracellular Heat Shock Protein 70
ECM	Extracellular Matrix
DAMP	Damage Associated Molecular Pattern
TLR4	Toll-Like Receptor 4
